# Validation of pharmacodynamic assays to evaluate the clinical efficacy of an antisense compound (AEG 35156) targeted to the X-linked inhibitor of apoptosis protein XIAP

**DOI:** 10.1038/sj.bjc.6602363

**Published:** 2005-02-01

**Authors:** J Cummings, T H Ward, E LaCasse, C Lefebvre, M St-Jean, J Durkin, M Ranson, C Dive

**Affiliations:** 1 Clinical and Experimental Pharmacology, Paterson Institute for Cancer Research, Christie Hospital NHS Trust, Wilmslow Road, Manchester M20 4BX, UK; 2 Aegera Oncology Inc., CHEO Research Institute, 401 Smyth Rd, Ottawa, Ontario, Canada K1H 8L1; 3 Department of Medical Oncology, Christie Hospital NHS Trust, Wilmslow Road, Manchester M20 4BX, UK

**Keywords:** XIAP, antisense, quantitative RT-PCR, Western blot analysis, M30-Apoptosense™ Elisa

## Abstract

The inhibitor of apoptosis protein, XIAP, is frequently overexpressed in chemoresistant human tumours. An antisense oligonucleotide (AEG 35156/GEM 640) that targets XIAP has recently entered phase I trials in the UK. Method validation data are presented on three pharmacodynamic assays that will be utilised during this trial. Quantitative RT-PCR was based on a Taqman assay and was confirmed to be specific for XIAP. Assay linearity extended over four orders of magnitude. MDA-MB-231/U6-E1 cells and clone X-G4 stably expressing an RNAi vector against XIAP were chosen as high and low XIAP expression quality controls (QCs). Within-day and between-day coefficients of variation (CVs) in precision for cycle threshold (CT) and delta CT values (employing GAPDH and beta 2 microglobulin as housekeepers) were always less than 10%. A Western blotting technique was validated using a GST–XIAP fusion protein as a standard and HeLa cells and SF268 (human glioblastoma) cells as high and low XIAP expression QCs. Specificity of the final choice of antibody for XIAP was evaluated by analysing a panel of cell lines including clone X-G4. The assay was linear over a 29-fold range of protein concentration and between-day precision was 29% for the low QC and 23% for the high QC when normalised to GAPDH. XIAP protein was also shown to be stable at −80°C for at least 60 days. M30-Apoptosense™ plasma Elisa detects a caspase-cleaved fragment of cytokeratin 18 (CK18), believed to be a surrogate marker for tumour cell apoptosis. Generation of an independent QC was achieved through the treatment of X-G4 cells with staurosporine and collection of media. Measurements on assay precision and kit-to-kit QC were always less than 10%. The M30 antigen (CK18-Asp^396^) was stable for 3 months at −80°C, while at 37°C it had a half-life of 80–100 h in healthy volunteer plasma. Results from the phase I trial are eagerly awaited.

The X-linked inhibitor of apoptosis, XIAP, is a member of a large family of proteins which share in common one or more structural motifs known as BIR domains (LaCasse *et al*, 1998; Liston *et al*, 2003). IAPs function to block cell death by binding to and inhibiting the action of caspases involved in the execution phase of apoptosis (Holcik and Korneluk, 2001; Holcik *et al*, 2001). XIAP is the most potent endogenous caspase inhibitor (Stennicke *et al*, 2002) and its overexpression results in a blockade of cell death arising from a number of different triggers including cytotoxic drugs, ionising radiation and growth factor deprivation (Holcik and Korneluk, 2001; Holcik *et al*, 2001). Thus, it can antagonise both the mitochondrial regulated (intrinsic) and death-receptor-mediated (extrinsic) apoptotic pathways (Hengartner, 2000). In clinical investigations, the protein has been shown to be overexpressed in a number of different tumours relative to normal tissue (Hofmann *et al*, 2002; Krajewska *et al*, 2003; Shiraki *et al*, 2003) and high expression is often associated with poor patient outcome (Tamm *et al*, 2000, 2004; Ramp *et al*, 2004; Yan *et al*, 2004) and resistance to chemotherapy (Parton *et al*, 2002).

Studies with knockout mice have shown that the absence of XIAP does not adversely affect the development of normal tissues (Harlin *et al*, 2001), whereas antisense knockdown (KD) of the protein in a non-small-cell lung cancer (NSCLC) xenograft (H460) produces significant antitumour activity (Hu *et al*, 2003). Furthermore, small-molecule derivatives of polyphenylurea, screened for efficacy in overcoming XIAP inhibition of caspase 3, have demonstrated *in vivo* antitumour activity against human prostate and colon cancer xenografts in the absence of significant toxicity to normal tissues (Schimmer *et al*, 2004). Thus, XIAP may represent a novel and tumour-selective therapeutic target for anticancer drug design (Huang *et al*, 2004).

Recently, a second-generation 19-mer antisense chimeric oligonucleotide targeting XIAP, constructed as a mixed backbone of chemically modified DNA/RNA nucleotides (denoted AEG 35156/GEM 640), has entered Phase I clinical evaluation at two different centres in the United Kingdom. An integral component of this clinical trial will be that pharmacodynamic (PD) studies are performed on patient-derived samples (plasma, peripheral blood mononuclear cells and tumour biopsies) in order to provide evidence of target KD. Laboratory studies that support clinical trials are being subjected increasingly to more stringent regulatory requirements, especially with the publication of the European Directive on Clinical Trials (Fontaine and Rosengren, 2001) as a Statutory Instrument (1031, HMSO) in the UK from May 2004. This paper describes the validation of three of the assays that will be employed during the phase I trial of AEG 35156/GEM 640.

## MATERIALS AND METHODS

### Reagents

Staurosporine was from the Sigma Chemical Company (Poole, England) and caspase inhibitor I (z-VAD) was from Calbiochem (Darmstadt, Germany). Custom-synthesised PCR primers for XIAP and RNase-free DNase were from Qiagen (Valencia, CA, USA). An XIAP-specific Taqman probe was obtained from IDT Inc. (Coralville, IO, USA). Taqman EZ reverse transcriptase (RT)–PCR core reagents kit, PCR primer pairs and gene-specific Taqman reagents for GADPH, cyclophilin A, beta 2 microglobulin, 18S rRNA and Tata-binding protein were all from Applied Biosystems Inc. (ABI, Foster City, CA, USA). GST–XIAP fusion protein was either produced in house (Aegera) or obtained from Alexis (ALEXIS Corporation LTD, Nottingham, UK). Monoclonal antibodies to XIAP were obtained as follows: hILP/XIAP clones 28 and 48 were from BD Biosciences (Pharmingen, San Diego, CA, USA) and anti-XIAP clone 2F1 was from MBL (Watertown, MA, USA). Anti-GAPDH monoclonal antibody (clone 6C5) was from Advanced ImmunoChemical Inc. (Long Beach, CA, USA). Goat anti-mouse secondary antibody was from Amersham (Arlington Heights, IL, USA) and geneticin was from Invitrogen Gibco BRL (Carlsbad, CA, USA). BSA protein standard was from Pierce (Rockford, IL, USA). M30-Apoptosense™ 96-well kits for the determination of cleaved cytokeratin (CK) 18 were from PEVIVA (Bromma, Sweden). All other reagents and chemicals were of the highest grade available commercially. Water was purified and deionised in a Millipore Elix 3 system (Millipore, Watford, England).

### Cell lines

MDA-MB-231/X-G4 human breast cancer cells stably transfected with a vector containing an siRNA to XIAP and parental MDA-MB-231/U6-E1 cells stably transfected with only the U6 promoter were generated in house (McManus *et al*, 2004). HeLa human cervical cancer cells were from the American Type Culture Collection (Manassas, VA, USA) and SF268 human glioblastoma cells were from the University of California in San Francisco Brain Tumour Bank (San Francisco, CA, USA). Cell lines were cultured in RPMI 1640 medium (BE12-167F, Cambrex Bio Science, Verviers, Belgium) containing 1% glutamine, and 10% foetal bovine serum (Harlan Sera-Lab, Loughborough, England) at 37°C in a humidified atmosphere of 5% CO_2_ in air. In the case of the X-G4 clone and parental U6-E1 cells, geneticin (G1397, Sigma) at a concentration of 300 *μ*g ml^−1^ was supplemented to media to maintain selection of transfected cells.

### Preparation of cancer cell pellets as quality control (QC) samples for both quantitative (q)RT-PCR and Western blotting

In order to act as QC samples and a surrogate matrix for tumour tissue, cancer cell lines were prepared as solid pellets from a stock culture and aliquoted into individual tubes according to the following procedure. Cells were grown to near confluence, then the medium was aspirated. The cell monolayers were washed twice with phosphate-buffered saline (PBS), pH 7.4, and detached according to a standard trypsinisation protocol. A volume of 5–10 ml of media was added and each cell suspension was transferred to a universal screw top container and centrifuged at 300 **g** for 5 min. The supernatants were discarded, the cell pellets resuspended in 20 ml of ice-cold PBS and centrifuged at 300 **g** for 5 min. Finally, the supernatants were discarded and the pellets stored at −80°C prior to analysis by qRT-PCR or Western blot analysis (WBA).

### XIAP qRT-PCR assay method

qRT-PCR was performed essentially as described in detail (Fong *et al*, 2000; McKinnon *et al*, 2002; Hu *et al*, 2003). In brief, after cell lysis RNA was extracted from cell pellets using the RNeasy mini kit (Qiagen) and then treated with RNase-free DNase. A pCI-plasmid containing XIAP cDNA, which was employed as a standard, was purified from transformed *Escherichia coli* using the HiSpeed plasmid maxi kit (Qiagen). Quantitation of extracted RNA and evaluation of purity was performed by UV spectrophotometry at 260 nm and by measuring absorbance ratios at 260 *vs* 280 nm. The quantity and quality of purified DNA were verified by spectrophotometry, as well as by agarose gel electrophoresis and ethidium bromide staining. For each cell line, 25 ng of total RNA was employed and was reverse-transcribed and PCR amplified utilising the Taqman EZ RT–PCR kit (ABI). XIAP primers and probes were used at concentrations of 600 and 200 nM, respectively. The thermal cycling conditions for the RT step were as follows: 50°C for 2 min, 60°C for 30 min and 95°C for 5 min, then followed by 45 cycles of PCR at 94°C for 20 s and 60°C for 1 min per cycle. All the RT–PCR steps were performed using an ABI Prism 7700 Sequence Detector (ABI), and quantitated using the cycle threshold (CT) method. XIAP RNA levels were also normalised to two housekeeping genes (delta CT method), and were also occasionally compared to another QC sample acting as a reference point (delta–delta CT).

### Western blot analysis of XIAP

Western blotting of cancer cell pellets was conducted essentially as described previously in detail (Hu *et al*, 2003). Pellets were homogenised in ice-cold lysis buffer consisting of 50 mM Tris–HCL, pH 7, 150 mM NaCl, 1 mM EDTA, 1 mM sodium fluoride, 1 mM vanadate, 1% (v v^−1^) Nonidet P-40 and 0.25% sodium deoxycholate in a cocktail of protease inhibitors (leupeptin, aprotinin and PMSF). Protein content of cell lysates was determined by the Bio-Rad assay, standardised with bovine serum albumin (Bio-Rad Laboratories, Hercules, CA, USA). Equal amounts of protein (10 *μ*g lane^−1^) were subjected to electrophoresis and separated on 12 or 4–15% gradient SDS–polyacrylamide gels and then transferred to Immobilon-P transfer membranes (Mandel, Guelph, Canada) overnight at 4°C in the presence of a blocking buffer containing 5% milk powder. The membranes were incubated for 2 h with one of the three different primary antibodies (clones 28 and 48 and 2F1) diluted 1 : 2000 in 1% milk powder buffer, washed and then further incubated for 1 h with the appropriate HRP-conjugated secondary antibody at a dilution of 1 : 2000 in 1% milk powder buffer. GAPDH was selected as the housekeeper protein to control for loading. Finally, proteins were detected using enhanced chemiluminescence (ECL, Amersham Pharmacia Biotech, Little Chalfont, England) and visualised after exposure to a Kodak autoradiography film. Scanning densitometry was performed to quantitate band intensities by volume/area integration (Molecular Dynamics, Sunnyvale, CA, USA).

### M30-Apoptosense™ Elisa

The M30-Apoptosense™ Elisa assay is a commercially available kit based on a 96-well plate format and was operated according to the manufacturer's instructions. In brief, 25 *μ*l of sample (standard, blank, QC, or culture medium) was added to each well, which was coated with a mouse monoclonal catcher antibody that binds to an epitope on CK18 (M6 antibody). Simultaneously, 75 *μ*l of HRP-conjugated monoclonal antibody (M30) solution was added to act as the detecting antibody by binding to the caspase cleavage site (neo-epitope (NE)) at the N-terminus of the Asp^396^ fragment of CK18 (Leers *et al*, 1999). Samples were then incubated for 4 h at room temperature with constant shaking, after which excess unbound conjugate was removed by addition of the wash solution, times five. Colour development was achieved by the addition of 200 *μ*l of 3,3′,5,5′-tetramethyl-benzidine solution, followed by an incubation for 20 min in the dark. The reaction was stopped by the addition of 50 *μ*l of 1.0 M sulphuric acid and absorbance was finally measured in a microplate reader at 450 nm. By plotting a standard curve of known concentrations of M30 antigen *vs* absorbance, the amount of antigen in the QCs and unknown samples was calculated by interpolation.

## RESULTS AND DISCUSSION

Three different methodologies have been selected for validation, each of which will be employed as a PD assay during a phase I trial to assess the efficacy of an antisense oligonucleotide AEG 35156/GEM 640 targeting the inhibitor of apoptosis protein XIAP. Current CR-UK policy regarding laboratory investigations that support clinical trials requires that a greater degree of method validation is performed depending on the order of priority designated to the PD assay (CR-UK policy document, 2001). These standards are likely to become even more exacting with the introduction of the EU directive on clinical trials 2001/20/EU (Fontaine and Rosengren, 2001) as a legal requirement in the UK (Statutory Instrument 1031, HMSO) from 1 May 2004. Since traditional end points such as bone marrow toxicity, developed for phase I evaluation of the toxicity of nonselective DNA-damaging cytotoxic drugs, may be less relevant to the newer molecular targeted therapies such as antisense, greater emphasis is now placed on measurement of biological responses (i.e. PD assays) as potential trial end points (Workman, 2003). However, many of the PD methods that measure putative changes in the expression of macromolecular drug targets are by their nature semiquantitative, and internationally accepted guidelines for the validation of such assays are less clear (Shah *et al*, 2000; Miller *et al*, 2001).

In the context of the present work, qRT-PCR was designated as the PD assay of primary importance, with the Western blotting technique being secondary to it. The M30-Apoptosense™ plasma Elisa is a surrogate assay for apoptosis occurring in the tumour (Biven *et al*, 2003; Ueno *et al*, 2003), which by itself is only potentially a secondary effect of antisense treatment, and was therefore considered a tertiary assay.

### qRT-PCR of XIAP mRNA in QC cell lines pellets

As a consequence of qRT-PCR being designated as the primary PD assay, the validation plan focused on several key elements: incorporation of an XIAP standard for calibration and determination of dynamic range; confirmation of the specificity of the commercially available PCR primers and Taqman probe for XIAP; identification of two internal control housekeeping genes and use of both high and low XIAP as QC samples.

**Figure 1:**
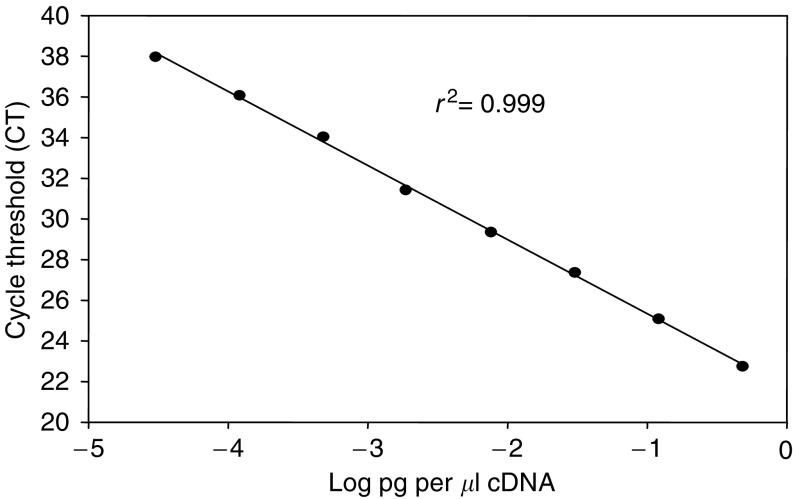
Dynamic range of a qRT-PCR method for XIAP evaluated using a purified pCI plasmid containing full-length human XIAP cDNA as the template.

A plasmid (pCI) containing human cDNA to XIAP was investigated as the standard and Figure 1 illustrates that the assay was linear (mean regression correlation coefficient (*r*^2^) value of 0.999±0.001 standard deviation (s.d., *n*=6) over a dynamic range of four orders of magnitude of DNA concentration. Limit of detection was defined as 0.00012 pg cDNA equivalents or a CT value of 38. However, it was observed that in blank no template control wells a significant CT value of 35 or above was occasionally recorded, which was eliminated in the absence of the cDNA calibration curve. It was concluded that the presence of high concentrations of cDNA on the plate was responsible for crosscontamination, and all subsequent validation experiments were based solely on the analysis of the precision of the QC samples. Thus, the plasmid XIAP standard was used only to establish the dynamic range of the assay. The lack of the calibration curve would define the assay as semiquantitative (Miller *et al*, 2001). Nevertheless, due to the inherent robustness of qRT-PCR as a quantitative technique, workers have suggested that comparable results can be obtained without the need for a calibration curve (Peirson *et al*, 2003).

The specificity of the commercially available Taqman probe and PCR primers for XIAP was confirmed in a series of preliminary experiments (data not shown) summarised as follows: a single and correct-sized amplicon was visualised on an acrylamide gel; the assay did not amplify mouse or rat XIAP orthologs; the signal was not destroyed by DNAse prior to the RT step, but was destroyed post RT step; the signal was eliminated by RNAse treatment prior to RT step but not post RT step and no signal was detected in no template controls.

A total of five different genes were investigated in order to identify two of them as internal control housekeepers: GADPH, cyclophilin A, beta 2 microglobulin, 18S rRNA and Tata-binding protein. GAPDH produced an almost parallel response to XIAP in the high-QC cell line, when different amounts of RNA were assayed, with a comparison of slope values equal to 0.0302, where 0 reflects a perfect match and 1 represents no correspondence. Of the other four genes, only beta 2 microglobulin achieved an acceptable parallel response (comparison of slopes=0.378). These two genes were taken forward as the two housekeepers in a series of precision experiments performed with the high and low XIAP expression QCs (see Table 1 for within-day and Table 2 for between-day). Either utilising the two housekeeper genes for delta CT measurements or the CT value of XIAP on its own, precision was always less than 5%, representing almost one order of magnitude less variability than normally expected in a PD assay of a macromolecule (Miller *et al*, 2001), but in line with recent validation reports for qRT-PCR methods the determination of transcripts of the Wilms tumour suppressor gene (WT1) (Dumur *et al*, 2002) and the bcr–abl oncogene (Jones *et al*, 2003).

**Figure 2:**
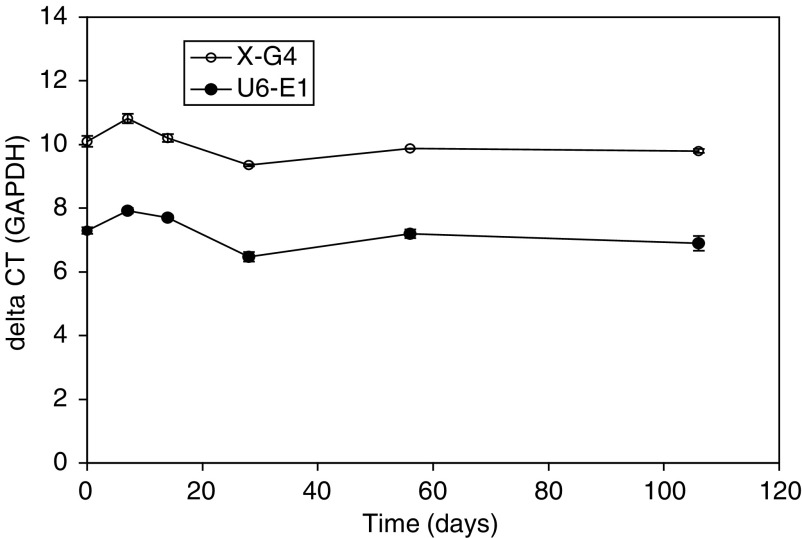
Stability of XIAP mRNA determined by qRT-PCR in replicate pellets of the high and low XIAP expression QC cell lines stored at −80°C over 106 days. At the time intervals indicated, three replicates were removed from the freezer and analysed. Each time point represents the mean±s.d. Results are expressed relative to GAPDH as delta CT values.

Stability of XIAP mRNA was determined by sequential analysis of triplicate cell pellets of the two different control cell lines (low and high XIAP expressers) over a period of 106 days. While values for XIAP mRNA determined by qRT-PCR fluctuated by as much as 10% both in an upwards and downwards direction, by day 106 of the incubation there was no significant difference in delta CT compared to time zero (Figure 2).

Many may consider immunohistochemistry (IHC) as the ideal primary PD assay technique to detect protein KD and thus evaluate an antisense therapy, rather than qRT-PCR. IHC has been applied in numerous previous reports in the analysis of XIAP expression in human tumours (Tamm *et al*, 2000; Ferreira *et al*, 2001; Bilim *et al*, 2003; Kashkar *et al*, 2003; Krajewska *et al*, 2003). However, almost without exception in these studies, the specificity of the technique was not evaluated using an XIAP-deficient or null matrix, such as the low QC used in the present study, the X-G4 clone stably transfected with a vector containing an siRNA to XIAP. In addition, it is clear that IHC – at best – yields only a three- to four-fold dynamic range of staining intensity (Bilim *et al*, 2003; Kashkar *et al*, 2003; Krajewska *et al*, 2003), which is unlikely to conclusively detect a modest level of KD (30–40%) that may nevertheless be sufficient to tip the balance between anti- and proapoptotic signalling in favour of cell death. The increased dynamic range, high sensitivity, consistency and robustness of the qRT-PCR assay reported in this paper were therefore given preference to the obvious benefit of IHC of being to visualise between tumour and nontumour cells in biopsies. This decision was strengthened by the fact that the majority of samples that will be collected during the phase I trial of AEG 35156/GEM 640 will be surrogate nontumour tissues, where the advantage of IHC is not relevant. While issues of dynamic range and consistency of staining may not be insurmountable obstacles to validating IHC, it is the specificity of the technique that raises most concern. Indeed, with the advantage of tissue from genetically engineered XIAP-deficient mice, we have observed significant staining, and therefore a lack of specificity, with a number of commercially available antibodies and with multiple staining protocols (unpublished observations).

### Western blot analysis of XIAP in QC cell line pellets

WBA has been employed to characterise the effect of antisense treatment on the expression levels of a number of antiapoptotic proteins, including Bcl-2 and Bcl-xL as well as XIAP (Jansen *et al*, 2000; Hayward *et al*, 2003; Hu *et al*, 2003). From the perspective of validation, two essential resources are required before a method can be judged as quantitative: (1) a purified standard of the protein of interest (XIAP) homologous to that of the endogenous protein and (2) a supply of blank control matrix similar to that of the patient samples, but minus the protein of interest (Miller *et al*, 2001). In the present study, an XIAP–GST fusion protein was used as the protein standard to yield XIAP concentrations in protein equivalents. This standard was added to 10 *μ*g of protein extract prepared from X-G4 cells stably expressing an RNAi with virtually undetectable XIAP protein to act as a surrogate matrix. In addition, XIAP protein levels were quantitated as a ratio to GAPDH, used as housekeeping protein to control for equal protein loading. The validation strategy concentrated on evaluating specificity, dynamic range, reproducibility and sample stability.

**Figure 3:**
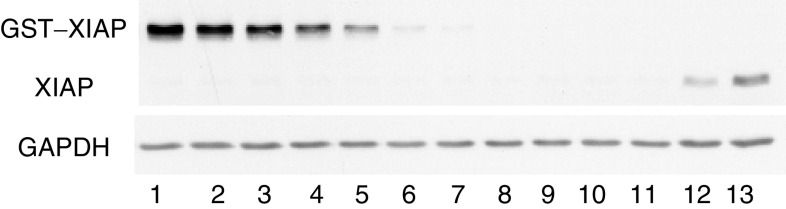
Calibration of a Western blotting technique for the determination of XIAP using a GST–XIAP protein standard. The fusion protein diluted in MDA-MB-231/X-G4 lysate was analysed within a concentration range of 0.06–135 pg *μ*g^−1^. Lanes are identified as follows: GST–XIAP (pg *μ*g^−1^) at 135, lane 1; 110, lane 2; 80, lane 3; 55, lane 4; 28, lane 5; 5.5, lane 6; 2.8, lane 7; 0.55, lane 8; 0.28, lane 9 and 0.06, lane 10. Also included on the blot are the XIAP-deficient cell line MDA-MB-231/X-G4, lane 11; the low QC cell line SF268, lane 12 and the high-QC cell line HeLa, lane 13. The blot was also probed for GAPDH, which acted as a housekeeper protein.

The main model to test the specificity of the antibody and the Western blotting technique was the XIAP-deficient cell line clone X-G4 (McManus *et al*, 2004). In addition, the presence of a single band corresponding to the molecular weight of the protein at 55 kDa in the parental cell line (U6-E1) was an absolute requirement, as well as an equivalent band in the two QC cell lines (HeLa and SF268). Of the three antibodies studied (see Materials and Methods), clone 28 produced the best results, satisfying the criteria set above, and was adopted for all future validation experiments (see lanes 11–13 in Figure 3).

The assay was demonstrated to exhibit a 29-fold linear dynamic range in protein concentration from 2.8 to 80 pg GST–XIAP per *μ*g matrix (Figure 3), with an average *r*^2^ value of 0.998±0.001 s.d., *n*=7. By interpolation, the low QC cell lines contained (approximately, see below for precision) 10 pg GST–XIAP equivalents per *μ*g protein and the high QC contained (approximately, see below for precision) 30 pg GST–XIAP equivalents per *μ*g protein (Figure 3).

**Figure 4:**
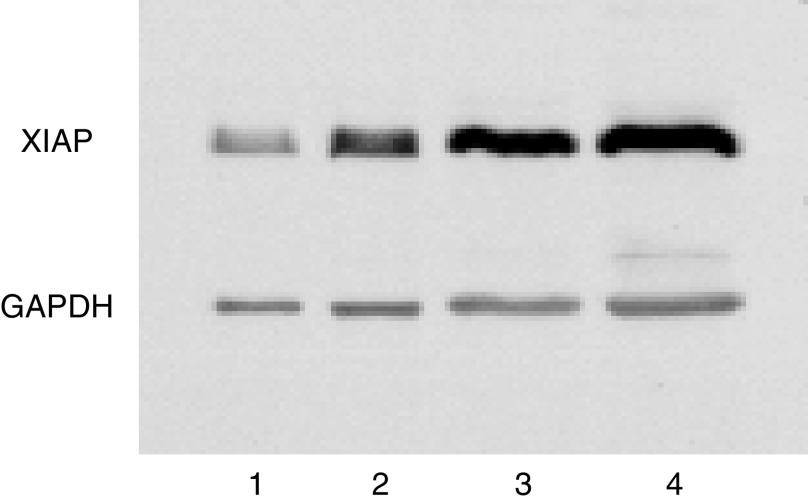
Western blot analysis of XIAP in peripheral blood mononuclear cells harvested from a healthy volunteer. Each lane was assayed at a different level of total protein loading. Lanes are identified as follows: 1, 5 *μ*g; 2, 10 *μ*g; 3, 20 *μ*g and 4, 40 *μ*g. The blot was also probed for GAPDH, which acted as a housekeeper protein.

Within-day and between-day precision data are presented for the low- and high-QC samples in Tables 3 and 4, respectively, and ranged from 9.1 to 12.8% for within-day and from 22.8 to 33.5% for between-day, even when normalised to GADPH. However, these values are in keeping with the expected norm for Western blot analysis (Miller *et al*, 2001). Figure 4 shows a typical Western blot analysis of peripheral blood mononuclear cells isolated from a healthy volunteer, analysed at four different protein-loading levels, together with GADPH as the housekeeper gene.

**Figure 5:**
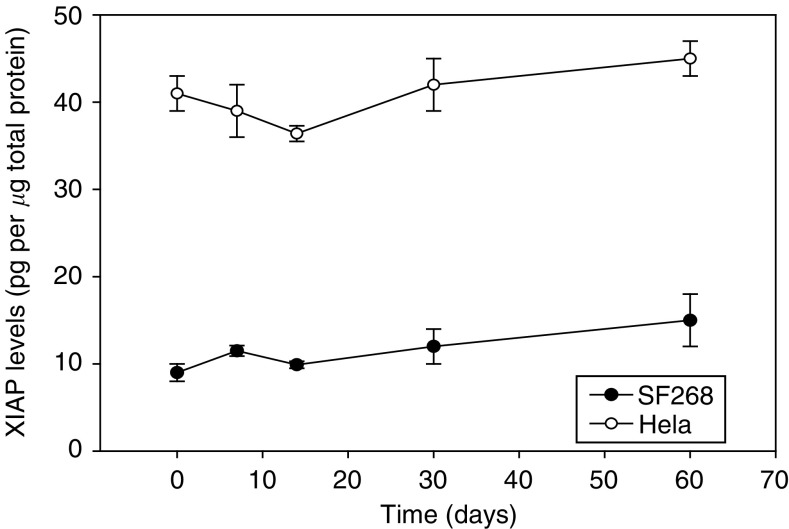
Stability of XIAP protein determined by Western blot in replicate pellets of the high and low XIAP expression QC cell lines stored at −80°C over 60 days. At the time intervals indicated, three replicates were removed from the freezer and analysed. Each time point represents the mean±s.d.

Stability of XIAP protein was determined by sequential analysis of triplicate cell pellets of the two different control cell lines (low and high XIAP expressers) over a period of 60 days. Results showed that no significant changes in XIAP protein equivalents (Figure 5) or XIAP/GAPDH ratios occurred.

### Detection of caspase-cleaved CK18 (CK-ASP^396^ NE) in QC samples by the M30-Apoptosense™ Elisa assay

Each M30-Apoptosense™ kit includes a five-point calibration curve (including a blank) extending over an antigen concentration range of 50–1000 U l^−1^ and high- and low-QC samples of certified antigen concentration (circa 750 and 100 U l^−1^). The focus of the validation plan was to generate independent positive and negative QC samples, in order to perform a more extensive evaluation of precision, conduct kit-to-kit QC and follow the stability of the antigen when stored at −80°C and exposed to 37°C. Independent QCs were generated by incubation of MDA-MB-231/X-G4 cells with 100 nM staurosporine for 48 h. After centrifugation, media were assayed to determine the level of antigen. Finally, media were pooled, diluted appropriately and individually aliquoted (150 positive and 100 negative aliquots) to yield a positive independent control with an antigen value of (circa) 600 U l^−1^. The aliquots of conditioned media were stored at −80°C.

**Figure 6:**
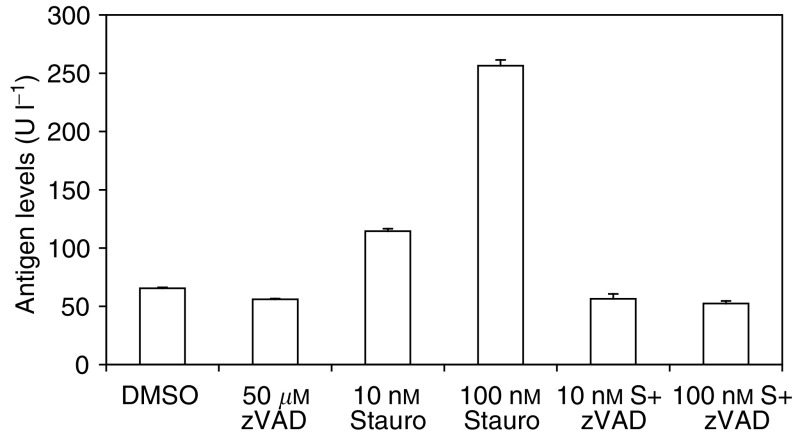
Effect of the treatment of MDA-MB-231/X-G4 cells with staurosporine plus or minus the general caspase inhibitor zVAD on the levels of CK18-asp^396^ NE antigen detected in the culture medium by the M30 Apoptosense Elisa assay. Cells were incubated for 24 h with either 10 or 100 nM staurosporine (Stauro, S) in the presence or absence of 50 *μ*M zVAD. Each bar represents the mean value±s.d., *n*=3.

In a separate study to confirm that the appearance of M30 antigen in the culture media was due to the induction of apoptosis, MDA-MB-231/X-G4 cells were treated with 10 or 100 nM staurosporine for 24 h in the presence or absence of the broad-spectrum caspase inhibitor z-VAD. Figure 6 shows that a 10-fold increase in concentration of staurosporine resulted in a four-fold increase in M30 antigen. The addition of 50 *μ*M z-VAD completely abolished the release of cleaved CK18 into media, both in the case of 10 and 100 nM staurosporine.

**Figure 7:**
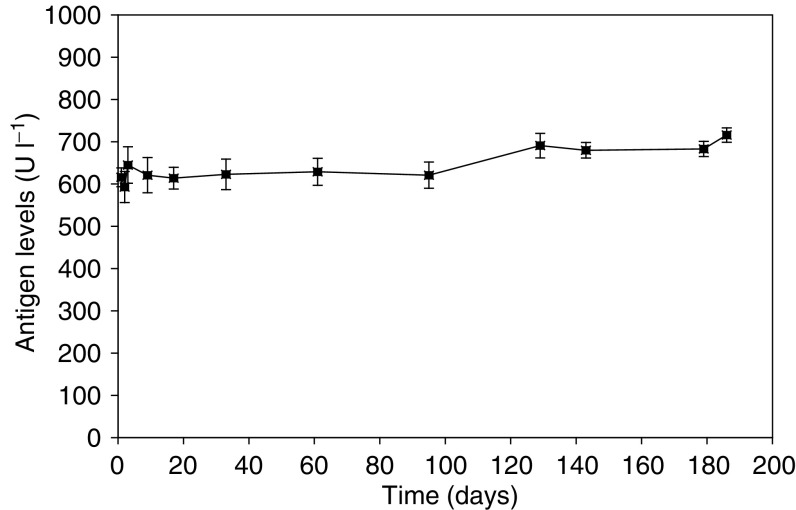
Stability of CK18-asp^396^ NE antigen in tissue culture medium stored at −80°C. Each time point represents the mean value±s.d., *n*=3.

**Figure 8:**
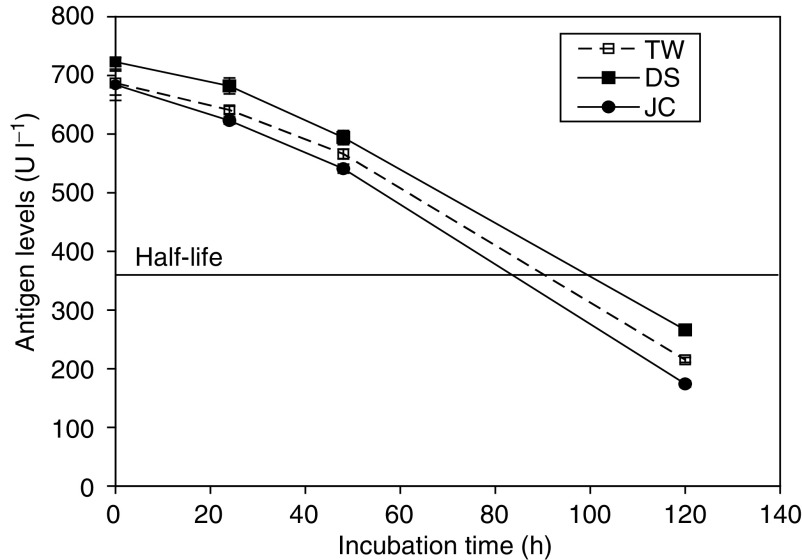
Stability of CK18-asp^396^ NE antigen in healthy volunteer plasma after incubation at 37°C in the dark. Blood was collected from three different subjects and each time point represents the mean value±s.d., *n*=3.

Using a series of nine different dilutions of the positive control, the calibration curve for the Elisa assay was demonstrated to follow the classic shape of a sigmoid plot with a value of *r*^2^ equalling 0.997, and a plateau occurring at antigen concentrations of 1000 U l^−1^ and above, the upper limit of the calibration curve supplied by the manufacturers. Typical within-day precision using eight replicates was 3.6% for the independent positive control and 6.7% for the negative control. Analyses performed on eight separate days over a 3-month period yielded a mean value of 620 U l^−1^ for the positive control, with a between-day precision of 2.4%. Kit-to-kit variations in the concentration of antigen determined in the positive control was 3.7% between kits 1 and 2, 4.8% between kits 2 and 3, and 1.5% between kits 3 and 4, well within the manufacturer's acceptance criteria of 10% (http://web.peviva.se/index.asp). The stability of the positive control QC samples when stored at −80°C was followed over a 6-month period (Figure 7). Values remained stable for 3 months, after which time there was a gradual and persistent increase from the plateau value of 620–716 U l^−1^, representing a 15.5% drift in concentration. The manufacturers recommend that plasma samples are stable for at least 6 months when stored at −80°C due to the robustness of the M30 antigen (Carr, 2000). In our studies, the QC samples consisted of RPMI media containing 10% serum and this may account for the difference. The M30 antigen (CK18-ASP^396^ NE) was relatively stable in volunteer plasma incubated at 37°C in the dark with a half-life of between 80 and 100 h (Figure 8).

M30 is a monoclonal antibody that recognises an NE mapped to positions 387–396, of a caspase-cleaved fragment of CK18, with a liberated C-terminus at the cleavage site of DALD-S (Leers *et al*, 1999). The antibody has been validated in the format of IHC for several years as a marker of apoptosis (marketed as M30 CytoDeath, Roche, Basel, Switzerland), and has been shown to be more robust than TUNEL and ISEL (Carr, 2000). It has been applied successfully in a number of patient studies to measure apoptosis (Kusama *et al*, 2000; Kadyrov *et al*, 2001; Leers *et al*, 2002; Mirzaie-Joniani *et al*, 2002; Rupa *et al*, 2003). More recently, the M30 antibody has been employed in the format of an Elisa assay for *in vitro* high throughput screening of apoptosis-inducing compounds against cancer cell lines and for the analysis of serum or plasma from patients as a surrogate marker for tumour cell apoptosis (Biven *et al*, 2003; Ueno *et al*, 2003). In this format, the assay relies on the fact that apoptotic cells release cleaved CK18 into the culture medium or circulation of patients. Since CK18 is believed to be expressed only in cells of epithelial origin, theoretically the assay should not be subjected to interference by bone marrow cell death.

In summary, validation strategies and data are presented on the performance of three PD assays commonly used in early clinical trials of new anticancer drugs using cancer cell pellets as a surrogate tissue for patient tumour biopsies. Although these assays are tailored for the analysis of an antisense therapy targeting XIAP, it is hoped that the general principles of method validation presented may have broader applicability to other new agents requiring PD end point assays during early-phase clinical evaluation.

## Figures and Tables

**Table 1:** Within-day precision in the measurement of XIAP by real-time RT–PCR in QC samples

	**Delta CT**		**CT XIAP**	
**Eight replicates in 1 day**	**Average**	**CV (%)**	**Average**	**CV (%)**
GAPDH				
U6-E1 (high QC)	7.3	2.27	28.36	0.34
X-G4 (low QC)	10.1	1.75	31.23	0.58
B2M				
U6-E1 (high QC)	4.8	4.23	28.26	0.48
X-G4 (low QC)	7.85	3.2	31.22	0.59

**Table 2:** Between-day precision in the measurement of XIAP by real-time RT–PCR in QC samples

	**Delta CT**		**CT XIAP**	
**Five separate days of three replicates**	**Average**	**CV%**	**Average**	**CV%**
GAPDH				
U6-E1 (high QC)	7.36	4.32	28.20	0.40
X-G4 (low QC)	10.3	3.48	31.18	0.37
B2M				
U6-E1 (high QC)	4.68	7.3	28.42	0.72
X-G4 (low QC)	7.84	4.28	31.47	0.79

**Table 3:** Within-day precision in the measurement of XIAP by Western blot analysis in QC samples

	**XIAP/GAPDH ratio**		**XIAP minus background**	
**Eight replicates**	**Mean value (pg per *μ*g total protein)**	**Precision (CV%)**	**Mean value (pg per *μ*g total protein)**	**Precision (CV%)**
SF268 (low QC)	10.3	11.2	12.3	9.1
HeLa (high QC)	28.1	12.8	28.9	11.0

**Table 4:** Between-day precision in the measurement of XIAP by Western blot analysis in QC samples

	**XIAP/GAPDH ratio**		**XIAP minus background**	
**Five different days, three replicates**	**Mean value (pg per *μ*g total protein)**	**Precision (CV%)**	**Mean value (pg *μ*g total protein)**	**Precision (CV%)**
SF268 (low QC)	8.0	28.7	8.6	33.5
HeLa (high QC)	30.1	22.8	34.1	23.6
